# Supplementary material for: Mental health status and quality of life in elderly patients with coronary heart disease
Source: PeerJ. 2021 Feb 17;9:e10903. doi: 10.7717/peerj.10903 (PMC7896500; doi:10.7717/peerj.10903)
Supplement: Supplemental Information 2 [file peerj-09-10903-s002.docx]

**Supplementary Table 1.** **Compare the scores of two scales in elderly CHD patients with** **primary hypertension and without primary hypertension**

| **Variables** | **Number (%)** | **Primary hypertension**  **(N=171;79.2%)** | **No primary hypertension**  **(N=45;20.8%)** | ***p value*** |
| --- | --- | --- | --- | --- |
| Average positive factors (SCL-90) | 216(100%) | 26.18 (14.88) | 20.69 (9.54) | 0.003 |
| Somatization | 216(100%) | 2.09 (0.76) | 1.90 (0.66) | 0.122 |
| Obsessive-compulsive | 216(100%) | 1.83 (0.53) | 1.72 (0.39) | 0.128 |
| Interpersonal sensitivity | 216(100%) | 1.41 (0.48) | 1.21 (0.32) | 0.001 |
| Depression | 216(100%) | 1.77 (0.62) | 1.52 (0.30) | <0.001 |
| Anxiety | 216(100%) | 1.57 (0.49) | 1.41 (0.32) | 0.011 |
| Hostility | 216(100%) | 1.41 (0.42) | 1.29 (0.43) | 0.073 |
| Phobic anxiety | 216(100%) | 1.46 (0.58) | 1.17 (0.24) | <0.001 |
| Paranoid ideation | 216(100%) | 1.29 (0.39) | 1.19 (0.28) | 0.046 |
| Psychoticism | 216(100%) | 1.42 (0.43) | 1.24 (0.32) | 0.002 |
| WHOQOL-BREF Physical | 216(100%) | 12.43 (2.82) | 13.58 (2.08) | 0.003 |
| WHOQOL-BREF Psychological | 216(100%) | 13.38 (2.42) | 13.94 (1.94) | 0.155 |
| WHOQOL-BREF Social | 216(100%) | 14.37 (2.06) | 14.58 (1.56) | 0.462 |
| WHOQOL-BREF Environmental | 216(100%) | 15.20 (1.75) | 15.48 (1.75) | 0.343 |

Data were shown as mean (SD). Continuous variables used independent sample t test.
