# Supplementary material for: Mental health status and quality of life in elderly patients with coronary heart disease
Source: PeerJ. 2021 Feb 17;9:e10903. doi: 10.7717/peerj.10903 (PMC7896500; doi:10.7717/peerj.10903)
Supplement: Supplemental Information 3 [file peerj-09-10903-s003.docx]

**Supplementary Table 2.** **Compare the scores of two scales in elderly CHD patients with** **type 2 DM and without type 2 DM**

| **Variables** | **Number (%)** | **Type 2 DM**  **(N=83;38.4%)** | **No Type 2 DM**  **(N=133;61.6%)** | ***p value*** |
| --- | --- | --- | --- | --- |
| Average positive factors (SCL-90) | 216(100%) | 29.54 (14.97) | 22.23 (12.79) | <0.001 |
| Somatization | 216(100%) | 2.32 (0.80) | 1.89 (0.66) | <0.001 |
| Obsessive-compulsive | 216(100%) | 1.91 (0.51) | 1.75 (0.50) | 0.024 |
| Interpersonal sensitivity | 216(100%) | 1.48 (0.49) | 1.30 (0.42) | 0.005 |
| Depression | 216(100%) | 1.90 (0.61) | 1.60 (0.53) | <0.001 |
| Anxiety | 216(100%) | 1.65 (0.53) | 1.46 (0.40) | 0.009 |
| Hostility | 216(100%) | 1.47 (0.43) | 1.33 (0.41) | 0.024 |
| Phobic anxiety | 216(100%) | 1.56 (0.62) | 1.30 (0.46) | 0.002 |
| Paranoid ideation | 216(100%) | 1.34 (0.40) | 1.22 (0.34) | 0.033 |
| Psychoticism | 216(100%) | 1.50 (0.46) | 1.31 (0.36) | 0.002 |
| WHOQOL-BREF Physical | 216(100%) | 11.74 (2.90) | 13.25 (2.44) | <0.001 |
| WHOQOL-BREF Psychological | 216(100%) | 12.93 (2.42) | 13.85 (2.22) | 0.005 |
| WHOQOL-BREF Social | 216(100%) | 14.09 (2.04) | 14.62 (1.90) | 0.053 |
| WHOQOL-BREF Environmental | 216(100%) | 15.03 (1.68) | 15.40 (1.79) | 0.133 |

Data were shown as mean (SD). Continuous variables used independent sample t test.
