# Supplementary material for: Mental health status and quality of life in elderly patients with coronary heart disease
Source: PeerJ. 2021 Feb 17;9:e10903. doi: 10.7717/peerj.10903 (PMC7896500; doi:10.7717/peerj.10903)
Supplement: Supplemental Information 4 [file peerj-09-10903-s004.docx]

**Supplementary Table 3. Compare the scores of two scales in elderly CHD patients with stroke and without stroke**

| **Variables** | **Number (%)** | **Stroke**  **(N=50；23.1%)** | **No Stroke**  **(N=166；76.9%)** | ***p value*** |
| --- | --- | --- | --- | --- |
| Average positive factors (SCL-90) | 216(100%) | 33.24 (15.66) | 22.57 (12.63) | <0.001 |
| Somatization | 216(100%) | 2.40 (0.85) | 1.95 (0.68) | 0.001 |
| Obsessive-compulsive | 216(100%) | 2.07 (0.56) | 1.73 (0.47) | <0.001 |
| Interpersonal sensitivity | 216(100%) | 1.58 (0.57) | 1.30 (0.40) | 0.002 |
| Depression | 216(100%) | 2.08 (0.72) | 1.61 (0.48) | <0.001 |
| Anxiety | 216(100%) | 1.69 (0.53) | 1.49 (0.43) | 0.007 |
| Hostility | 216(100%) | 1.49 (0.40) | 1.36 (0.43) | 0.055 |
| Phobic anxiety | 216(100%) | 1.71 (0.62) | 1.31 (0.48) | <0.001 |
| Paranoid ideation | 216(100%) | 1.45 (0.45) | 1.21 (0.32) | 0.001 |
| Psychoticism | 216(100%) | 1.65 (0.51) | 1.30 (0.34) | <0.001 |
| WHOQOL-BREF Physical | 216(100%) | 11.31 (2.96) | 13.08 (2.51) | <0.001 |
| WHOQOL-BREF Psychological | 216(100%) | 12.84 (2.72) | 13.70 (2.18) | 0.022 |
| WHOQOL-BREF Social | 216(100%) | 14.25 (2.34) | 14.46 (1.85) | 0.509 |
| WHOQOL-BREF Environmental | 216(100%) | 14.86 (1.83) | 15.38 (1.71) | 0.067 |

Data were shown as mean (SD). Continuous variables used independent sample t test.
