# Supplementary material for: Mental health status and quality of life in elderly patients with coronary heart disease
Source: PeerJ. 2021 Feb 17;9:e10903. doi: 10.7717/peerj.10903 (PMC7896500; doi:10.7717/peerj.10903)
Supplement: Supplemental Information 5 [file peerj-09-10903-s005.docx]

**Mental health status and quality of life in elderly patients with** **coronary heart disease**

**Statistical analysis**

Data were shown as number (%) or as mean (standard deviation). The t test was used for the continuous variables. The associations were examined using multiple stepwise linear regression analyses, and variables including age, sex, BMI, marital status, primary hypertension, type 2 diabetes mellitus and stroke were entered into the multiple stepwise linear regression. We used multiple stepwise linear regression analyses to generate significant fit models. The coefficients and 95% confidence interval (CI) for each significant variable were determined. A *p*-value of <0.05 was considered statistically significant. Statistical analyses were performed using IBM SPSS 24.0.

For the manuscript, at first, there were 14 models to establish, and one model (dependent variable: the scores of WHOQOL-BREF social domain) suggested that no independent variable had significant influence on dependent variables, therefore, we showed 13 models in the following results. For 13 models, the regression tolerance of each model is greater than 0.1, and there was no multicollinearity.

**Independent variables:** age, sex (reference: male), BMI, marital status (reference: widowed or bachelor), primary hypertension (reference: no primary hypertension), type 2 diabetes mellitus (reference: no type 2 diabetes mellitus) and stroke (reference: no stroke);

**Dependent variables:** average positive factors, somatization, obsessive-compulsive, interpersonal sensitivity, depression, anxiety, hostility, phobic anxiety, paranoid ideation, psychoticism, physical, psychological, social, environment.

**Abbreviation in the following results:** independent variables: Marital-marital status; Hypertension- primary hypertension; Diabetes- type 2 diabetes mellitus; Dependent variable: APF - Average positive factors; OC -obsessive-compulsive; IS-Interpersonal sensitivity; Phobic-Phobic anxiety; PI-Paranoid ideation.

**Model 1:** Dependent Variable-APF (Average positive factors)

REGRESSION

/DESCRIPTIVES MEAN STDDEV CORR SIG N

/MISSING LISTWISE

/STATISTICS COEFF OUTS CI (95) R ANOVA COLLIN TOL ZPP

/CRITERIA=PIN (.05) POUT (.10)

/NOORIGIN

/DEPENDENT APF

/METHOD=STEPWISE Sex Marital BMI Age Hypertension Diabetes Stroke

/SCATTERPLOT= (*ZRESID, *ZPRED) (*ZPRED, APF)

/RESIDUALS HISTOGRAM(ZRESID) NORMPROB(ZRESID)

/CASEWISE PLOT(ZRESID) OUTLIERS (3)

/SAVE PRED COOK LEVER SRESID SDRESID.

| **Model Summary** | | | | |
| --- | --- | --- | --- | --- |
| Model | R | R Square | Adjusted R Square | Std. Error of the Estimate |
| 1 | .326^a^ | .106 | .102 | 13.35440 |
| 2 | .437^b^ | .191 | .184 | 12.73573 |
| 3 | .503^c^ | .253 | .242 | 12.26796 |
| 4 | .541^d^ | .292 | .279 | 11.96858 |
| a. Predictors: (Constant), Sex | | | | |
| b. Predictors: (Constant), Sex, Stroke | | | | |
| c. Predictors: (Constant), Sex, Stroke, Marital | | | | |
| d. Predictors: (Constant), Sex, Stroke, Marital, Diabetes | | | | |
| e. Dependent Variable: APF | | | | |

| Model | | Unstandardized Coefficients | | Sig. | 95.0% Confidence Interval for B | | Collinearity Statistics | |
| --- | --- | --- | --- | --- | --- | --- | --- | --- |
|  |  | B | Std. Error |  | Lower Bound | Upper Bound | Tolerance | VIF |
| 1 | (Constant) | 19.906 | 1.363 | .000 | 17.220 | 22.593 |  |  |
|  | Sex | 9.235 | 1.829 | .000 | 5.631 | 12.840 | 1.000 | 1.000 |
| 2 | (Constant) | 18.079 | 1.356 | .000 | 15.406 | 20.753 |  |  |
|  | Sex | 8.464 | 1.752 | .000 | 5.011 | 11.917 | .991 | 1.009 |
|  | Stroke | 9.744 | 2.064 | .000 | 5.676 | 13.811 | .991 | 1.009 |
| 3 | (Constant) | 37.078 | 4.719 | .000 | 27.775 | 46.380 |  |  |
|  | Sex | 8.812 | 1.689 | .000 | 5.482 | 12.142 | .989 | 1.011 |
|  | Stroke | 9.510 | 1.989 | .000 | 5.590 | 13.430 | .991 | 1.010 |
|  | Marital | -19.779 | 4.721 | .000 | -29.085 | -10.473 | .997 | 1.003 |
| 4 | (Constant) | 35.851 | 4.618 | .000 | 26.748 | 44.954 |  |  |
|  | Sex | 8.355 | 1.653 | .000 | 5.096 | 11.614 | .982 | 1.018 |
|  | Stroke | 8.680 | 1.955 | .000 | 4.826 | 12.534 | .975 | 1.025 |
|  | Marital | -20.357 | 4.609 | .000 | -29.442 | -11.272 | .996 | 1.004 |
|  | Diabetes | 5.809 | 1.696 | .001 | 2.467 | 9.152 | .975 | 1.026 |

**Model 2**: Dependent Variable- Somatization

REGRESSION

/DESCRIPTIVES MEAN STDDEV CORR SIG N

/MISSING LISTWISE

/STATISTICS COEFF OUTS CI (95) R ANOVA COLLIN TOL ZPP

/CRITERIA=PIN (.05) POUT (.10)

/NOORIGIN

/DEPENDENT Somatization

/METHOD=STEPWISE Sex Marital BMI Age Hypertension Diabetes Stroke

/SCATTERPLOT= (*ZRESID, *ZPRED) (*ZPRED, Somatization)

/RESIDUALS HISTOGRAM(ZRESID) NORMPROB(ZRESID)

/CASEWISE PLOT(ZRESID) OUTLIERS (3)

/SAVE PRED COOK LEVER SRESID SDRESID.

| Model Summary | | | | |
| --- | --- | --- | --- | --- |
| Model | R | R Square | Adjusted R Square | Std. Error of the Estimate |
| 1 | .319a | .102 | .098 | .70912 |
| 2 | .405b | .164 | .156 | .68568 |
| 3 | .452c | .204 | .193 | .67074 |
| a. Predictors: (Constant), Sex | | | | |
| b. Predictors: (Constant), Sex, Diabetes | | | | |
| c. Predictors: (Constant), Sex, Diabetes, Stroke | | | | |
| d. Dependent Variable: Somatization | | | | |

| Model | | Unstandardized Coefficients | | Sig. | 95.0% Confidence Interval for B | | Collinearity Statistics | |
| --- | --- | --- | --- | --- | --- | --- | --- | --- |
|  |  | B | Std. Error |  | Lower Bound | Upper Bound | Tolerance | VIF |
| 1 | (Constant) | 1.787 | .072 | .000 | 1.644 | 1.930 |  |  |
|  | Sex | .478 | .097 | .000 | .287 | .670 | 1.000 | 1.000 |
| 2 | (Constant) | 1.659 | .077 | .000 | 1.507 | 1.811 |  |  |
|  | Sex | .443 | .094 | .000 | .257 | .629 | .991 | 1.009 |
|  | Diabetes | .384 | .096 | .000 | .194 | .574 | .991 | 1.009 |
| 3 | (Constant) | 1.605 | .077 | .000 | 1.453 | 1.757 |  |  |
|  | Sex | .418 | .093 | .000 | .236 | .601 | .985 | 1.016 |
|  | Diabetes | .346 | .095 | .000 | .159 | .533 | .976 | 1.024 |
|  | Stroke | .356 | .110 | .001 | .141 | .572 | .976 | 1.024 |

**Model 3**: Dependent Variable- OC (Obsessive-compulsive)

REGRESSION

/DESCRIPTIVES MEAN STDDEV CORR SIG N

/MISSING LISTWISE

/STATISTICS COEFF OUTS CI (95) R ANOVA COLLIN TOL ZPP

/CRITERIA=PIN (.05) POUT (.10)

/NOORIGIN

/DEPENDENT OC

/METHOD=STEPWISE Sex Marital BMI Age Hypertension Diabetes Stroke

/SCATTERPLOT= (*ZRESID, *ZPRED) (*ZPRED, OC)

/RESIDUALS HISTOGRAM(ZRESID) NORMPROB(ZRESID)

/CASEWISE PLOT(ZRESID) OUTLIERS (3)

/SAVE PRED COOK LEVER SRESID SDRESID.

| Model Summary | | | | |
| --- | --- | --- | --- | --- |
| Model | R | R Square | Adjusted R Square | Std. Error of the Estimate |
| 1 | .287a | .082 | .078 | .48826 |
| 2 | .385b | .148 | .140 | .47158 |
| 3 | .459c | .211 | .199 | .45497 |
| a. Predictors: (Constant), Stroke | | | | |
| b. Predictors: (Constant), Stroke, Sex | | | | |
| c. Predictors: (Constant), Stroke, Sex, Marital | | | | |
| d. Dependent Variable: OC | | | | |

| Model | | Unstandardized Coefficients | | Sig. | 95.0% Confidence Interval for B | | Collinearity Statistics | |
| --- | --- | --- | --- | --- | --- | --- | --- | --- |
|  |  | B | Std. Error |  | Lower Bound | Upper Bound | Tolerance | VIF |
| 1 | (Constant) | 1.729 | .038 | .000 | 1.654 | 1.804 |  |  |
|  | Stroke | .345 | .079 | .000 | .190 | .500 | 1.000 | 1.000 |
| 2 | (Constant) | 1.590 | .050 | .000 | 1.491 | 1.689 |  |  |
|  | Stroke | .316 | .076 | .000 | .166 | .467 | .991 | 1.009 |
|  | Sex | .263 | .065 | .000 | .135 | .391 | .991 | 1.009 |
| 3 | (Constant) | 2.280 | .175 | .000 | 1.935 | 2.625 |  |  |
|  | Stroke | .308 | .074 | .000 | .162 | .453 | .991 | 1.010 |
|  | Sex | .275 | .063 | .000 | .152 | .399 | .989 | 1.011 |
|  | Marital | -.718 | .175 | .000 | -1.064 | -.373 | .997 | 1.003 |

**Model 4**: Dependent Variable- IS (Interpersonal sensitivity)

REGRESSION

/DESCRIPTIVES MEAN STDDEV CORR SIG N

/MISSING LISTWISE

/STATISTICS COEFF OUTS CI (95) R ANOVA COLLIN TOL ZPP

/CRITERIA=PIN (.05) POUT (.10)

/NOORIGIN

/DEPENDENT IS

/METHOD=STEPWISE Sex Marital BMI Age Hypertension Diabetes Stroke

/SCATTERPLOT= (*ZRESID, *ZPRED) (*ZPRED, IS)

/RESIDUALS HISTOGRAM(ZRESID) NORMPROB(ZRESID)

/CASEWISE PLOT(ZRESID) OUTLIERS (3)

/SAVE PRED COOK LEVER SRESID SDRESID.

| Model Summary | | | | |
| --- | --- | --- | --- | --- |
| Model | R | R Square | Adjusted R Square | Std. Error of the Estimate |
| 1 | .257a | .066 | .062 | .44232 |
| 2 | .314b | .099 | .090 | .43555 |
| 3 | .362c | .131 | .119 | .42856 |
| 4 | .396d | .157 | .141 | .42323 |
| a. Predictors: (Constant), Stroke | | | | |
| b. Predictors: (Constant), Stroke, Sex | | | | |
| c. Predictors: (Constant), Stroke, Sex, Marital | | | | |
| d. Predictors: (Constant), Stroke, Sex, Marital, Diabetes | | | | |
| e. Dependent Variable: IS | | | | |

| Model | | Unstandardized Coefficients | | Sig. | 95.0% Confidence Interval for B | | Collinearity Statistics | |
| --- | --- | --- | --- | --- | --- | --- | --- | --- |
|  |  | B | Std. Error |  | Lower Bound | Upper Bound | Tolerance | VIF |
| 1 | (Constant) | 1.303 | .034 | .000 | 1.235 | 1.370 |  |  |
|  | Stroke | .277 | .071 | .000 | .137 | .418 | 1.000 | 1.000 |
| 2 | (Constant) | 1.215 | .046 | .000 | 1.123 | 1.306 |  |  |
|  | Stroke | .259 | .071 | .000 | .120 | .398 | .991 | 1.009 |
|  | Sex | .166 | .060 | .006 | .048 | .284 | .991 | 1.009 |
| 3 | (Constant) | 1.663 | .165 | .000 | 1.338 | 1.988 |  |  |
|  | Stroke | .254 | .069 | .000 | .117 | .391 | .991 | 1.010 |
|  | Sex | .174 | .059 | .003 | .058 | .291 | .989 | 1.011 |
|  | Marital | -.467 | .165 | .005 | -.792 | -.142 | .997 | 1.003 |
| 4 | (Constant) | 1.631 | .163 | .000 | 1.309 | 1.953 |  |  |
|  | Stroke | .232 | .069 | .001 | .096 | .368 | .975 | 1.025 |
|  | Sex | .162 | .058 | .006 | .047 | .278 | .982 | 1.018 |
|  | Marital | -.482 | .163 | .003 | -.803 | -.160 | .996 | 1.004 |
|  | Diabetes | .151 | .060 | .012 | .033 | .270 | .975 | 1.026 |

**Model 5**: Dependent Variable- Depression

REGRESSION

/DESCRIPTIVES MEAN STDDEV CORR SIG N

/MISSING LISTWISE

/STATISTICS COEFF OUTS CI (95) R ANOVA COLLIN TOL ZPP

/CRITERIA=PIN (.05) POUT (.10)

/NOORIGIN

/DEPENDENT Depression

/METHOD=STEPWISE Sex Marital BMI Age Hypertension Diabetes Stroke

/SCATTERPLOT= (*ZRESID, *ZPRED) (*ZPRED, Depression)

/RESIDUALS HISTOGRAM(ZRESID) NORMPROB(ZRESID)

/CASEWISE PLOT(ZRESID) OUTLIERS (3)

/SAVE PRED COOK LEVER SRESID SDRESID.

| Model Summary | | | | |
| --- | --- | --- | --- | --- |
| Model | R | R Square | Adjusted R Square | Std. Error of the Estimate |
| 1 | .344a | .118 | .114 | .54595 |
| 2 | .416b | .173 | .166 | .52988 |
| 3 | .473c | .223 | .212 | .51487 |
| 4 | .513d | .263 | .249 | .50265 |
| a. Predictors: (Constant), Stroke | | | | |
| b. Predictors: (Constant), Stroke, Marital | | | | |
| c. Predictors: (Constant), Stroke, Marital, Sex | | | | |
| d. Predictors: (Constant), Stroke, Marital, Sex, Diabetes | | | | |
| e. Dependent Variable: Depression | | | | |

| Model | | Unstandardized Coefficients | | Sig. | 95.0% Confidence Interval for B | | Collinearity Statistics | |
| --- | --- | --- | --- | --- | --- | --- | --- | --- |
|  |  | B | Std. Error |  | Lower Bound | Upper Bound | Tolerance | VIF |
| 1 | (Constant) | 1.608 | .042 | .000 | 1.524 | 1.691 |  |  |
|  | Stroke | .472 | .088 | .000 | .299 | .646 | 1.000 | 1.000 |
| 2 | (Constant) | 2.352 | .202 | .000 | 1.954 | 2.749 |  |  |
|  | Stroke | .465 | .086 | .000 | .296 | .633 | .999 | 1.001 |
|  | Marital | -.767 | .204 | .000 | -1.168 | -.365 | .999 | 1.001 |
| 3 | (Constant) | 2.248 | .198 | .000 | 1.857 | 2.638 |  |  |
|  | Stroke | .436 | .083 | .000 | .271 | .600 | .991 | 1.010 |
|  | Marital | -.803 | .198 | .000 | -1.193 | -.412 | .997 | 1.003 |
|  | Sex | .261 | .071 | .000 | .122 | .401 | .989 | 1.011 |
| 4 | (Constant) | 2.197 | .194 | .000 | 1.815 | 2.579 |  |  |
|  | Stroke | .401 | .082 | .000 | .239 | .563 | .975 | 1.025 |
|  | Marital | -.827 | .194 | .000 | -1.208 | -.445 | .996 | 1.004 |
|  | Sex | .243 | .069 | .001 | .106 | .379 | .982 | 1.018 |
|  | Diabetes | .241 | .071 | .001 | .100 | .381 | .975 | 1.026 |

**Model 6**: Dependent Variable- Anxiety

REGRESSION

/DESCRIPTIVES MEAN STDDEV CORR SIG N

/MISSING LISTWISE

/STATISTICS COEFF OUTS CI (95) R ANOVA COLLIN TOL ZPP

/CRITERIA=PIN (.05) POUT (.10)

/NOORIGIN

/DEPENDENT Anxiety

/METHOD=STEPWISE Sex Marital BMI Age Hypertension Diabetes Stroke

/SCATTERPLOT= (*ZRESID, *ZPRED) (*ZPRED, Anxiety)

/RESIDUALS HISTOGRAM(ZRESID) NORMPROB(ZRESID)

/CASEWISE PLOT(ZRESID) OUTLIERS (3)

/SAVE PRED COOK LEVER SRESID SDRESID.

| Model Summary | | | | |
| --- | --- | --- | --- | --- |
| Model | R | R Square | Adjusted R Square | Std. Error of the Estimate |
| 1 | .277a | .077 | .072 | .44498 |
| 2 | .388b | .151 | .143 | .42779 |
| 3 | .426c | .181 | .170 | .42100 |
| 4 | .445d | .198 | .183 | .41764 |
| a. Predictors: (Constant), Sex | | | | |
| b. Predictors: (Constant), Sex, Marital | | | | |
| c. Predictors: (Constant), Sex, Marital, Diabetes | | | | |
| d. Predictors: (Constant), Sex, Marital, Diabetes, Stroke | | | | |
| e. Dependent Variable: Anxiety | | | | |

| Model | | Unstandardized Coefficients | | Sig. | 95.0% Confidence Interval for B | | Collinearity Statistics | |
| --- | --- | --- | --- | --- | --- | --- | --- | --- |
|  |  | B | Std. Error |  | Lower Bound | Upper Bound | Tolerance | VIF |
| 1 | (Constant) | 1.392 | .045 | .000 | 1.302 | 1.481 |  |  |
|  | Sex | .257 | .061 | .000 | .137 | .377 | 1.000 | 1.000 |
| 2 | (Constant) | 2.071 | .164 | .000 | 1.748 | 2.393 |  |  |
|  | Sex | .268 | .059 | .000 | .153 | .384 | .998 | 1.002 |
|  | Marital | -.709 | .165 | .000 | -1.033 | -.384 | .998 | 1.002 |
| 3 | (Constant) | 2.029 | .162 | .000 | 1.711 | 2.348 |  |  |
|  | Sex | .253 | .058 | .000 | .139 | .368 | .989 | 1.011 |
|  | Marital | -.724 | .162 | .000 | -1.043 | -.404 | .997 | 1.003 |
|  | Diabetes | .167 | .059 | .005 | .050 | .283 | .990 | 1.010 |
| 4 | (Constant) | 1.997 | .161 | .000 | 1.680 | 2.315 |  |  |
|  | Sex | .243 | .058 | .000 | .130 | .357 | .982 | 1.018 |
|  | Marital | -.713 | .161 | .000 | -1.030 | -.396 | .996 | 1.004 |
|  | Diabetes | .151 | .059 | .011 | .035 | .268 | .975 | 1.026 |
|  | Stroke | .143 | .068 | .037 | .009 | .278 | .975 | 1.025 |

**Model 7**: Dependent Variable- Hostility

REGRESSION

/DESCRIPTIVES MEAN STDDEV CORR SIG N

/MISSING LISTWISE

/STATISTICS COEFF OUTS CI (95) R ANOVA COLLIN TOL ZPP

/CRITERIA=PIN (.05) POUT (.10)

/NOORIGIN

/DEPENDENT Hostility

/METHOD=STEPWISE Sex Marital BMI Age Hypertension Diabetes Stroke

/SCATTERPLOT= (*ZRESID, *ZPRED) (*ZPRED, Hostility)

/RESIDUALS HISTOGRAM(ZRESID) NORMPROB(ZRESID)

/CASEWISE PLOT(ZRESID) OUTLIERS (3)

/SAVE PRED COOK LEVER SRESID SDRESID.

| Model Summary | | | | |
| --- | --- | --- | --- | --- |
| Model | R | R Square | Adjusted R Square | Std. Error of the Estimate |
| 1 | .153a | .023 | .019 | .41966 |
| 2 | .215b | .046 | .037 | .41574 |
| 3 | .267c | .071 | .058 | .41116 |
| 4 | .305d | .093 | .076 | .40725 |
| a. Predictors: (Constant), Diabetes | | | | |
| b. Predictors: (Constant), Diabetes, Age | | | | |
| c. Predictors: (Constant), Diabetes, Age, Stroke | | | | |
| d. Predictors: (Constant), Diabetes, Age, Stroke, Marital | | | | |
| e. Dependent Variable: Hostility | | | | |

| Model | | Unstandardized Coefficients | | Sig. | 95.0% Confidence Interval for B | | Collinearity Statistics | |
| --- | --- | --- | --- | --- | --- | --- | --- | --- |
|  |  | B | Std. Error |  | Lower Bound | Upper Bound | Tolerance | VIF |
| 1 | (Constant) | 1.335 | .036 | .000 | 1.263 | 1.407 |  |  |
|  | Diabetes | .133 | .059 | .024 | .017 | .249 | 1.000 | 1.000 |
| 2 | (Constant) | 1.884 | .247 | .000 | 1.397 | 2.370 |  |  |
|  | Diabetes | .140 | .058 | .017 | .025 | .255 | .997 | 1.003 |
|  | Age | -.008 | .003 | .026 | -.014 | -.001 | .997 | 1.003 |
| 3 | (Constant) | 2.017 | .250 | .000 | 1.523 | 2.510 |  |  |
|  | Diabetes | .123 | .058 | .035 | .008 | .237 | .983 | 1.018 |
|  | Age | -.010 | .004 | .005 | -.017 | -.003 | .923 | 1.083 |
|  | Stroke | .167 | .070 | .017 | .030 | .304 | .910 | 1.099 |
| 4 | (Constant) | 2.426 | .307 | .000 | 1.821 | 3.032 |  |  |
|  | Diabetes | .128 | .058 | .027 | .015 | .242 | .981 | 1.019 |
|  | Age | -.011 | .004 | .002 | -.018 | -.004 | .910 | 1.099 |
|  | Stroke | .168 | .069 | .016 | .032 | .304 | .910 | 1.099 |
|  | Marital | -.356 | .158 | .025 | -.667 | -.045 | .984 | 1.017 |

**Model 8**: Dependent Variable- Phobic (Phobic anxiety)

REGRESSION

/DESCRIPTIVES MEAN STDDEV CORR SIG N

/MISSING LISTWISE

/STATISTICS COEFF OUTS CI (95) R ANOVA COLLIN TOL ZPP

/CRITERIA=PIN (.05) POUT (.10)

/NOORIGIN

/DEPENDENT Phobic

/METHOD=STEPWISE Sex Marital BMI Age Hypertension Diabetes Stroke

/SCATTERPLOT= (*ZRESID, *ZPRED) (*ZPRED, Phobic)

/RESIDUALS HISTOGRAM(ZRESID) NORMPROB(ZRESID)

/CASEWISE PLOT(ZRESID) OUTLIERS (3)

/SAVE PRED COOK LEVER SRESID SDRESID.

| Model Summary | | | | |
| --- | --- | --- | --- | --- |
| Model | R | R Square | Adjusted R Square | Std. Error of the Estimate |
| 1 | .315a | .099 | .095 | .51617 |
| 2 | .377b | .142 | .134 | .50485 |
| 3 | .414c | .172 | .160 | .49721 |
| 4 | .445d | .198 | .183 | .49029 |
| 5 | .463e | .215 | .196 | .48645 |
| a. Predictors: (Constant), Stroke | | | | |
| b. Predictors: (Constant), Stroke, Sex | | | | |
| c. Predictors: (Constant), Stroke, Sex, Diabetes | | | | |
| d. Predictors: (Constant), Stroke, Sex, Diabetes, Marital | | | | |
| e. Predictors: (Constant), Stroke, Sex, Diabetes, Marital, Hypertension | | | | |
| f. Dependent Variable: Phobic | | | | |

| Model | | Unstandardized Coefficients | | Sig. | 95.0% Confidence Interval for B | | Collinearity Statistics | |
| --- | --- | --- | --- | --- | --- | --- | --- | --- |
|  |  | B | Std. Error |  | Lower Bound | Upper Bound | Tolerance | VIF |
| 1 | (Constant) | 1.308 | .040 | .000 | 1.229 | 1.387 |  |  |
|  | Stroke | .404 | .083 | .000 | .240 | .568 | 1.000 | 1.000 |
| 2 | (Constant) | 1.188 | .054 | .000 | 1.082 | 1.294 |  |  |
|  | Stroke | .379 | .082 | .000 | .218 | .540 | .991 | 1.009 |
|  | Sex | .227 | .069 | .001 | .090 | .364 | .991 | 1.009 |
| 3 | (Constant) | 1.129 | .057 | .000 | 1.016 | 1.241 |  |  |
|  | Stroke | .351 | .081 | .000 | .191 | .511 | .976 | 1.024 |
|  | Sex | .212 | .069 | .002 | .076 | .347 | .985 | 1.016 |
|  | Diabetes | .194 | .070 | .006 | .055 | .333 | .976 | 1.024 |
| 4 | (Constant) | 1.607 | .189 | .000 | 1.234 | 1.980 |  |  |
|  | Stroke | .344 | .080 | .000 | .187 | .502 | .975 | 1.025 |
|  | Sex | .220 | .068 | .001 | .086 | .353 | .982 | 1.018 |
|  | Diabetes | .201 | .069 | .004 | .064 | .338 | .975 | 1.026 |
|  | Marital | -.500 | .189 | .009 | -.873 | -.128 | .996 | 1.004 |
| 5 | (Constant) | 1.499 | .195 | .000 | 1.115 | 1.883 |  |  |
|  | Stroke | .320 | .080 | .000 | .162 | .479 | .955 | 1.047 |
|  | Sex | .215 | .067 | .002 | .083 | .348 | .981 | 1.019 |
|  | Diabetes | .165 | .071 | .021 | .025 | .305 | .917 | 1.090 |
|  | Marital | -.512 | .187 | .007 | -.881 | -.142 | .995 | 1.005 |
|  | Hypertension | .178 | .086 | .038 | .010 | .347 | .909 | 1.101 |

**Model 9**: Dependent Variable- PI (Paranoid ideation)

REGRESSION

/DESCRIPTIVES MEAN STDDEV CORR SIG N

/MISSING LISTWISE

/STATISTICS COEFF OUTS CI (95) R ANOVA COLLIN TOL ZPP

/CRITERIA=PIN (.05) POUT (.10)

/NOORIGIN

/DEPENDENT PI

/METHOD=STEPWISE Sex Marital BMI Age Hypertension Diabetes Stroke

/SCATTERPLOT= (*ZRESID, *ZPRED) (*ZPRED, PI)

/RESIDUALS HISTOGRAM(ZRESID) NORMPROB(ZRESID)

/CASEWISE PLOT(ZRESID) OUTLIERS (3)

/SAVE PRED COOK LEVER SRESID SDRESID.

| Model Summary | | | | |
| --- | --- | --- | --- | --- |
| Model | R | R Square | Adjusted R Square | Std. Error of the Estimate |
| 1 | .270a | .073 | .068 | .35565 |
| 2 | .376b | .141 | .133 | .34301 |
| 3 | .397c | .158 | .146 | .34054 |
| a. Predictors: (Constant), Marital | | | | |
| b. Predictors: (Constant), Marital, Stroke | | | | |
| c. Predictors: (Constant), Marital, Stroke, Diabetes | | | | |
| d. Dependent Variable: PI | | | | |

| Model | | Unstandardized Coefficients | | Sig. | 95.0% Confidence Interval for B | | Collinearity Statistics | |
| --- | --- | --- | --- | --- | --- | --- | --- | --- |
|  |  | B | Std. Error |  | Lower Bound | Upper Bound | Tolerance | VIF |
| 1 | (Constant) | 1.809 | .134 | .000 | 1.544 | 2.074 |  |  |
|  | Marital | -.560 | .137 | .000 | -.829 | -.290 | 1.000 | 1.000 |
| 2 | (Constant) | 1.743 | .131 | .000 | 1.486 | 2.001 |  |  |
|  | Marital | -.547 | .132 | .000 | -.807 | -.287 | .999 | 1.001 |
|  | Stroke | .229 | .055 | .000 | .120 | .338 | .999 | 1.001 |
| 3 | (Constant) | 1.720 | .130 | .000 | 1.463 | 1.976 |  |  |
|  | Marital | -.558 | .131 | .000 | -.816 | -.299 | .998 | 1.002 |
|  | Stroke | .214 | .055 | .000 | .105 | .323 | .982 | 1.018 |
|  | Diabetes | .097 | .048 | .044 | .003 | .192 | .981 | 1.019 |

**Model 10**: Dependent Variable- Psychoticism

REGRESSION

/DESCRIPTIVES MEAN STDDEV CORR SIG N

/MISSING LISTWISE

/STATISTICS COEFF OUTS CI (95) R ANOVA COLLIN TOL ZPP

/CRITERIA=PIN (.05) POUT (.10)

/NOORIGIN

/DEPENDENT Psychoticism

/METHOD=STEPWISE Sex Marital BMI Age Hypertension Diabetes Stroke

/SCATTERPLOT= (*ZRESID, *ZPRED) (*ZPRED, Psychoticism)

/RESIDUALS HISTOGRAM(ZRESID) NORMPROB(ZRESID)

/CASEWISE PLOT(ZRESID) OUTLIERS (3)

/SAVE PRED COOK LEVER SRESID SDRESID.

| Model Summary | | | | |
| --- | --- | --- | --- | --- |
| Model | R | R Square | Adjusted R Square | Std. Error of the Estimate |
| 1 | .360a | .130 | .126 | .38617 |
| 2 | .423b | .179 | .171 | .37610 |
| 3 | .470c | .221 | .210 | .36705 |
| 4 | .500d | .250 | .236 | .36098 |
| a. Predictors: (Constant), Stroke | | | | |
| b. Predictors: (Constant), Stroke, Sex | | | | |
| c. Predictors: (Constant), Stroke, Sex, Marital | | | | |
| d. Predictors: (Constant), Stroke, Sex, Marital, Diabetes | | | | |
| e. Dependent Variable: Psychoticism | | | | |

| Model | | Unstandardized Coefficients | | Sig. | 95.0% Confidence Interval for B | | Collinearity Statistics | |
| --- | --- | --- | --- | --- | --- | --- | --- | --- |
|  |  | B | Std. Error |  | Lower Bound | Upper Bound | Tolerance | VIF |
| 1 | (Constant) | 1.302 | .030 | .000 | 1.243 | 1.361 |  |  |
|  | Stroke | .352 | .062 | .000 | .229 | .475 | 1.000 | 1.000 |
| 2 | (Constant) | 1.204 | .040 | .000 | 1.125 | 1.283 |  |  |
|  | Stroke | .332 | .061 | .000 | .212 | .452 | .991 | 1.009 |
|  | Sex | .184 | .052 | .000 | .082 | .286 | .991 | 1.009 |
| 3 | (Constant) | 1.667 | .141 | .000 | 1.389 | 1.946 |  |  |
|  | Stroke | .326 | .059 | .000 | .209 | .444 | .991 | 1.010 |
|  | Sex | .192 | .051 | .000 | .093 | .292 | .989 | 1.011 |
|  | Marital | -.482 | .141 | .001 | -.760 | -.203 | .997 | 1.003 |
| 4 | (Constant) | 1.636 | .139 | .000 | 1.362 | 1.911 |  |  |
|  | Stroke | .305 | .059 | .000 | .189 | .422 | .975 | 1.025 |
|  | Sex | .181 | .050 | .000 | .082 | .279 | .982 | 1.018 |
|  | Marital | -.496 | .139 | .000 | -.770 | -.222 | .996 | 1.004 |
|  | Diabetes | .146 | .051 | .005 | .045 | .247 | .975 | 1.026 |

**Model 11**: Dependent Variable- Physical

REGRESSION

/DESCRIPTIVES MEAN STDDEV CORR SIG N

/MISSING LISTWISE

/STATISTICS COEFF OUTS CI (95) R ANOVA COLLIN TOL ZPP

/CRITERIA=PIN (.05) POUT (.10)

/NOORIGIN

/DEPENDENT Physical

/METHOD=STEPWISE Sex Marital BMI Age Hypertension Diabetes Stroke

/SCATTERPLOT= (*ZRESID, *ZPRED) (*ZPRED, Physical)

/RESIDUALS HISTOGRAM(ZRESID) NORMPROB(ZRESID)

/CASEWISE PLOT(ZRESID) OUTLIERS (3)

/SAVE PRED COOK LEVER SRESID SDRESID.

| Model Summary | | | | |
| --- | --- | --- | --- | --- |
| Model | R | R Square | Adjusted R Square | Std. Error of the Estimate |
| 1 | .275a | .076 | .072 | 2.61906 |
| 2 | .364b | .132 | .124 | 2.54361 |
| 3 | .424c | .180 | .168 | 2.47867 |
| 4 | .458d | .210 | .195 | 2.43854 |
| a. Predictors: (Constant), Stroke | | | | |
| b. Predictors: (Constant), Stroke, Diabetes | | | | |
| c. Predictors: (Constant), Stroke, Diabetes, Sex | | | | |
| d. Predictors: (Constant), Stroke, Diabetes, Sex, Marital | | | | |
| e. Dependent Variable: Physical | | | | |

| Model | | Unstandardized Coefficients | | Sig. | 95.0% Confidence Interval for B | | Collinearity Statistics | |
| --- | --- | --- | --- | --- | --- | --- | --- | --- |
|  |  | B | Std. Error |  | Lower Bound | Upper Bound | Tolerance | VIF |
| 1 | (Constant) | 13.079 | .203 | .000 | 12.678 | 13.480 |  |  |
|  | Stroke | -1.771 | .423 | .000 | -2.604 | -.938 | 1.000 | 1.000 |
| 2 | (Constant) | 13.546 | .234 | .000 | 13.085 | 14.007 |  |  |
|  | Stroke | -1.570 | .414 | .000 | -2.385 | -.754 | .983 | 1.017 |
|  | Diabetes | -1.337 | .359 | .000 | -2.045 | -.630 | .983 | 1.017 |
| 3 | (Constant) | 14.147 | .285 | .000 | 13.585 | 14.709 |  |  |
|  | Stroke | -1.453 | .405 | .000 | -2.251 | -.655 | .976 | 1.024 |
|  | Diabetes | -1.236 | .351 | .001 | -1.927 | -.544 | .976 | 1.024 |
|  | Sex | -1.200 | .342 | .001 | -1.874 | -.526 | .985 | 1.016 |
| 4 | (Constant) | 11.601 | .941 | .000 | 9.746 | 13.456 |  |  |
|  | Stroke | -1.416 | .398 | .000 | -2.202 | -.631 | .975 | 1.025 |
|  | Diabetes | -1.272 | .345 | .000 | -1.953 | -.591 | .975 | 1.026 |
|  | Sex | -1.244 | .337 | .000 | -1.908 | -.580 | .982 | 1.018 |
|  | Marital | 2.662 | .939 | .005 | .811 | 4.513 | .996 | 1.004 |

**Model 12**: Dependent Variable- Psychological

REGRESSION

/DESCRIPTIVES MEAN STDDEV CORR SIG N

/MISSING LISTWISE

/STATISTICS COEFF OUTS CI (95) R ANOVA COLLIN TOL ZPP

/CRITERIA=PIN (.05) POUT (.10)

/NOORIGIN

/DEPENDENT Psychological

/METHOD=STEPWISE Sex Marital BMI Age Hypertension Diabetes Stroke

/SCATTERPLOT= (*ZRESID, *ZPRED) (*ZPRED, Psychological)

/RESIDUALS HISTOGRAM(ZRESID) NORMPROB(ZRESID)

/CASEWISE PLOT(ZRESID) OUTLIERS (3)

/SAVE PRED COOK LEVER SRESID SDRESID.

| Model Summary | | | | |
| --- | --- | --- | --- | --- |
| Model | R | R Square | Adjusted R Square | Std. Error of the Estimate |
| 1 | .208a | .043 | .039 | 2.29248 |
| 2 | .271b | .073 | .065 | 2.26165 |
| 3 | .305c | .093 | .080 | 2.24290 |
| a. Predictors: (Constant), Sex | | | | |
| b. Predictors: (Constant), Sex, Diabetes | | | | |
| c. Predictors: (Constant), Sex, Diabetes, Marital | | | | |
| d. Dependent Variable: Psychological | | | | |

| Model | | Unstandardized Coefficients | | Sig. | 95.0% Confidence Interval for B | | Collinearity Statistics | |
| --- | --- | --- | --- | --- | --- | --- | --- | --- |
|  |  | B | Std. Error |  | Lower Bound | Upper Bound | Tolerance | VIF |
| 1 | (Constant) | 14.042 | .234 | .000 | 13.580 | 14.503 |  |  |
|  | Sex | -.978 | .314 | .002 | -1.596 | -.359 | 1.000 | 1.000 |
| 2 | (Constant) | 14.319 | .254 | .000 | 13.819 | 14.820 |  |  |
|  | Sex | -.901 | .311 | .004 | -1.514 | -.288 | .991 | 1.009 |
|  | Diabetes | -.833 | .318 | .009 | -1.460 | -.207 | .991 | 1.009 |
| 3 | (Constant) | 12.557 | .861 | .000 | 10.859 | 14.255 |  |  |
|  | Sex | -.930 | .309 | .003 | -1.539 | -.321 | .989 | 1.011 |
|  | Diabetes | -.855 | .315 | .007 | -1.477 | -.234 | .990 | 1.010 |
|  | Marital | 1.847 | .863 | .034 | .145 | 3.548 | .997 | 1.003 |

**Model 13**: Dependent Variable- Social

No independent variables in this model.

**Model 14**: Dependent Variable- Environment (Environment)

REGRESSION

/DESCRIPTIVES MEAN STDDEV CORR SIG N

/MISSING LISTWISE

/STATISTICS COEFF OUTS CI (95) R ANOVA COLLIN TOL ZPP

/CRITERIA=PIN (.05) POUT (.10)

/NOORIGIN

/DEPENDENT Environment

/METHOD=STEPWISE Sex Marital BMI Age Hypertension Diabetes Stroke

/SCATTERPLOT= (*ZRESID, *ZPRED) (*ZPRED, Environment)

/RESIDUALS HISTOGRAM(ZRESID) NORMPROB(ZRESID)

/CASEWISE PLOT(ZRESID) OUTLIERS (3)

/SAVE PRED COOK LEVER SRESID SDRESID.

| Model Summary | | | | |
| --- | --- | --- | --- | --- |
| Model | R | R Square | Adjusted R Square | Std. Error of the Estimate |
| 1 | .178a | .032 | .027 | 1.72686 |
| a. Predictors: (Constant), Sex | | | | |
| b. Dependent Variable: Environment | | | | |

| Model | | Unstandardized Coefficients | | Sig. | 95.0% Confidence Interval for B | | Collinearity Statistics | |
| --- | --- | --- | --- | --- | --- | --- | --- | --- |
|  |  | B | Std. Error |  | Lower Bound | Upper Bound | Tolerance | VIF |
| 1 | (Constant) | 15.604 | .176 | .000 | 15.257 | 15.952 |  |  |
|  | Sex | -.625 | .236 | .009 | -1.091 | -.159 | 1.000 | 1.000 |
