# Supplementary material for: Mental health status and quality of life in elderly patients with coronary heart disease
Source: PeerJ. 2021 Feb 17;9:e10903. doi: 10.7717/peerj.10903 (PMC7896500; doi:10.7717/peerj.10903)
Supplement: Supplemental Information 6 [file peerj-09-10903-s006.docx]

**Supplementary Table 4. Description of Variables of elderly CHD patients in the study**

| **Variable** | **Definition** |
| --- | --- |
| Age (years) | Age in Years, continuous variable |
| Sex | Sex (Male / Female), categorical variable; reference: male. |
| Height (cm) | Continuous variable |
| Weight (kg) | Continuous variable |
| BMI | Body Mass Index (kg/m^2), continuous variable |
| Marital status | Marital status (Married / Widowed or bachelor), categorical variable; reference: widowed or bachelor. |
| Primary hypertension | Primary hypertension Status (Yes / No), categorical variable; reference: no primary hypertension. |
| Type 2 DM | Type 2 diabetes mellitus (Yes / No), categorical variable; reference: no type 2 diabetes mellitus. |
| Stroke | History of stroke (Yes / No), categorical variable; reference: no stroke. |

Abbreviation:

BMI - body mass index

Type 2 DM- type 2 diabetes mellitus
